# Employee and customer handling of nicotine-containing e-liquids in vape shops

**DOI:** 10.18332/tpc/67295

**Published:** 2017-01-20

**Authors:** Robert Garcia, Jon Patrick Allem, Lourdes Baezconde-Garbanati, Jennifer Beth Unger, Steve Sussman

**Affiliations:** a University of Southern California, Los Angeles, USA

**Keywords:** Vape Shops, Nicotine handling, E-liquids, Electronic cigarettes, Vaping

## Abstract

**INTRODUCTION:**

Vape shops sell electronic cigarettes and related products such as e-liquids, which may contain nicotine. Direct contact with nicotine can lead to adverse health effects, and few regulations exist on how nicotine is handled in vape shops. This study examined how customers and employees come into contact with, and handle, nicotine-containing e-liquids in vape shops with the goal of informing potential future regulation of nicotine handling in vape shops.

**METHODS:**

Data were collected from 77 vape shops in the Los Angeles basin. Characteristics of the shops were documented by employee interviews and in store observations. Data collection was focused on shops located in areas with high concentrations of communities of interest; 20 shops from African-American communities, 17 from Hispanic communities, 18 from Korean communities, and 22 from non-Hispanic White communities.

**RESULTS:**

Half of the vape shops allowed customers to sample e-liquids with nicotine. Most of the shops (83%) provided self-service sampling stations for customers. A majority of shop employees (72%) reported that spills of e-liquids containing nicotine had occurred in the past. While 64% of the shops provided safety equipment, only 34% provided equipment for proper nicotine handling. Furthermore, 62% of shop employees reported handling nicotine without gloves or other safety equipment.

**CONCLUSIONS:**

Regulation on the handling of nicotine by customers and vape shop employees is important to prevent unsafe practices and subsequent injury. The frequent occurrence of spills and limited availability of safety equipment in vape shops highlights the need for the creation and enforcement of regulations to protect employees and customers. Appropriate safety training and equipment should be provided to employees to prevent accidental exposure to nicotine. Information on ways to safely handle nicotine should be communicated to vape shop employees and customers.

## INTRODUCTION

The rise in the popularity of electronic cigarettes (e-cigarettes) has led to the emergence of new retail outlets, vape shops, which supply rechargeable e-cigarettes, custom blended e-liquids and accessories^1–4^. Vape shop employees and customers can be exposed to nicotine-containing e-liquids, which can lead to adverse health effects (diarrhea, nausea, vomiting)^3,5,6^. The nicotine concentration in most e-liquids range from 0 to 36 mg/mL^7,8^ but some 10 mL bottles can contain up to 720 mg of nicotine^3,9^. The toxicity of nicotine has been documented^10^. Symptoms of nicotine poisoning include nausea, dizziness, vomiting, along with eye irritation, and severe cases can lead to death especially among children^10–16^. The current accepted oral fatal dose is 1 mg per kg of body weight for an adult but there have been cases of people ingesting higher amounts and surviving^17^.

The federal government provides guidelines for handling nicotine through the Occupational Safety and Health Administration (OSHA), but these guidelines have mainly centered on the safety of tobacco leaf harvesting and processing plants^18^. Recent research demonstrated that calls to poison control centers regarding nicotine exposure from e-cigarettes have increased markedly over the past four years^12^. Calls commonly dealt with eye and skin irritation as well as ingestion especially among children^13^.

While studies on nicotine poisoning have focused on the occupational hazard in both the harvesting of tobacco leaves and as a pesticide in horticultural settings,^11,14–16^ there is little information on nicotine-related hazards in vape shops^19^. It is important to address the customer and employee handling of nicotine-containing e-liquids in vape shops to prevent poisoning through skin or eye absorption, and inhalation. This study examined how customers and employees of vape shops handled nicotine-containing e-liquids. Findings should inform public and occupational health agencies on how to address the handling and packaging (e.g. warning labels, bottles that can reduce accidental spillage, the use of safety equipment for the handling of nicotine) of nicotine-containing e-liquids in vape shops to reduce the risks for employees and customers. Findings should also inform communication strategies about the potential harmful effects and risks posed by handling e-liquids.

## METHODS

Data were collected from a study on vape shops in the greater Los Angeles area^1,2,4^. This study examined characteristics of the shops through employee interviews and store observations. The vape shops were identified through a Yelp search^2^ and were included if they only sold e-cigarettes and related products and did not sell other tobacco products like combustible cigarettes (see Sussman et al., 2014 for more details). Communities of interest were identified through publicly available US Census data and mapped out by racial/ethnic composition. We focused on these racial/ethnic groups because they are representative of the diversity in the Los Angeles area and have distinct tobacco use profiles^20^. Data collectors attempted to contact 104 shops of which 17 were found to be out of business, 4 declined to participate, 4 were tobacco shops (which also sold e-cigarettes), 1 was a hookah lounge/vape shop, and 1 was a distributor. The remaining 77 shops were surveyed, observed, and included in analyses.

The final sample included 20 shops from African-American communities, 17 from Hispanic communities, 18 from Korean communities, and 22 non-Hispanic White communities. Allem et al4 found significant differences in vape shop employees’ attitudes and use of tobacco products between ethnic communities providing further support for exploring differences across communities.

### Data Collection

Data collectors approached shop employees to conduct the interview and shop observation. The interview was administered to shop employees (clerks, managers, or shop owners) based on availability. The employee who participated in the interview received a $50 gift card for their time and for allowing the data collector to conduct the shop observation. Two data collectors visited each shop so that one could conduct the shop observation while the other administered the interview. Data were collected from June 19, 2014 to December 8, 2014.

### Measures

#### Employee Interview

The interview asked participants about their age, gender, ethnicity, spoken languages, position at the vape shop, and length of employment. Participants were asked if they permitted customers to sample nicotine-containing e-liquids and at what concentrations. Participants were asked about how custom blends of nicotine-containing e-liquids were prepared, and whether employees or customers made the custom blends or if all e-liquids sold came pre-packaged. Participants were asked about the length and type of training they received about nicotine handling and whether they were told about the toxicity of nicotine and any other related dangers. Questions also pertained to whether there had ever been a spill of nicotine-containing e-liquid, and whether participants had ever touched nicotine-containing e-liquids directly without gloves, and if the shop provided safety equipment (gloves and goggles).

#### Shop Observation Form

The shop observation form assessed prices, types of e-cigarettes, e-liquids, promotional materials, merchandise, and rebuild equipment on display, as well as evidence of e-liquid spills.

### Analysis

Chi-square tests were conducted to determine if nicotine handling, and the availability of pre-mixed e-liquids, varied across communities. Chi-square tests were also conducted to examine whether stores that allowed customers to mix their blends of nicotine-containing e-liquids were less likely to use safety equipment (i.e. gloves, safety eyewear, masks), and whether employee nicotine handling practices differed by the level of training they received.

## RESULTS

Vape shop employees were primarily male (86%) and the mean age was 27.9 years (Table 1). About 25% of participants reported being the owner, 39% as manager, 33% a clerk, and 4% other (usually an owner/manager or owner/clerk). The mean length of employment was 11.2 months (sd=5.0).

All retailers sold rechargeable e-cigarettes, while 17% sold disposable types. Respondents indicated that they sold products with various amounts of nicotine concentrations including: 0 mg/mL (99%), 3 mg/mL (76%), 6 mg/mL (96%), 12 mg/mL (97 %), 18 mg/mL (86%), 20 mg/mL (4%), 24 mg/mL (42%), and other, higher concentrations going up to 36mg/mL (40.8%). Table 2 presents data on the handling of nicotine-containing e-liquids. All of the shops reported that they permit their customers to take free trial puffs. 50% of the vape shop employees reported that they permit customers to have free samples of nicotine-containing e-cigarettes. During the shop observation we also found that 83% had self-service stations or free samples of e-liquids visible and accessible to customers. Most of the shops (85%) gave samples with a nicotine concentration between 6–10 mg/mL. About 44% of vape shop employees reported providing samples with lower concentrations (1–3 mg/mL), while 18% reported that their samples had nicotine concentrations between 11 and 30 mg/mL. When asked about the mixing of nicotine-containing e-liquids at the shop, 32% reported that employees could do the mixing, and 24% reported that customers were allowed to mix the e-liquids. More than half (56%) of the employees reported that they only sold e-liquids that were mixed before arriving at the shop. Only 8% of employees responded yes to all three scenarios (pre-mixed, employee mixing, and customer mixed). Having pre-mixed solutions in the shops differed by community (p<0.05) with 30% of shops in non-Hispanic white communities, 24% in African American, 23% in Korean, and 22% in Hispanic communities offering pre-mixed solutions. Employees of shops that were located in non-Hispanic White communities (30%) were significantly more likely (Fishers, p<0.05) to report having pre-mixed solutions in their shops when compared to shops in African American (24%), Korean (23%), and Hispanic (22%) communities.

72% of employees reported that there had been spills of nicotine-containing e-liquid, and 35% of the shops that reported spills had evidence of spills during the in-store observation along with 26% of the shops that reported no spills during the employee interview. Data collectors reported that most (88%) of the observed spills were on shop counters or near the self-service areas.

### Employee Training

Only 17% of the participants indicated that they received training on how to handle nicotine and the information they received focused on the use of safety equipment (gloves), exercising caution when handling e-juices, and the importance of washing any areas of the skin exposed to nicotine. Shops that reported training varied significantly across ethnic communities (Fishers, p<0.05), with 30% of shops in African American communities, 28% in Hispanic communities, 41% in Korean communities, and18% in non-Hispanic White communities reporting they receive such training. Only 20% of vape shop employees indicated that they were informed of the dangers of handling nicotine.

### Safety Equipment & Clean-up Procedures

The majority of shop employees (84%) reported that safety equipment such as gloves or goggles were provided for the employees. Having evidence of spills differed by the availability of safety equipment (Fisher’s p=0.05), with 35% of shops that provided safety equipment and 18% of shops that did not provide safety equipment observed to have evidence of spills. Most shops (81%) however, reported that safety equipment was for e-cigarette rebuilds (e.g., drilling extra holes in vape pens), while 56% reported that it was provided for nicotine handling. About 65% of employees reported that they touch nicotine-containing e-liquids directly without gloves. The ANOVA test found that reports of employees touching nicotine varied significantly by length of employment of those interviewed (F=8.44, p<0.01) with those who had worked less than a year at the shop more likely to report touching nicotine without gloves. 29% of participants indicated that were told to wear gloves while cleaning up and 16% did not mention any cleanup protocol in their response.

## DISCUSSION

The present study is the first to document vape shops’ practices for handling nicotine-containing e-liquids, as a potential occupational hazard. A strength of this study is that the observational component allowed us to directly observe events in the vape shop rather than relying only on employees’ self-reports, which could be subject to social desirability bias. The frequent occurrence of spills and limited availability of safety equipment in vape shops highlights the need for the creation and enforcement of regulations to protect employees and customers. Regulations could include, but are not limited to, smaller bottle openings to decrease chance of spills, wearing protective gloves, along with clear and obvious warning labels. While it would take large amounts of nicotine-containing e-liquids to be fatal to an adult, there are still concerns with other adverse health effects (e.g., nausea, dizziness, eye irritation) that could occur^13^. Some of the employees interviewed received no training at all in handling of Nicotine containing products, and most were not informed of the dangers of nicotine-containing e-liquids. Findings provide directions for public health professionals, labor unions, health protection agencies, and regulatory bodies on the need for educational materials for vape shops, e.g., instructions on proper handling, clean up, and practices for customer access to nicotine-containing e-liquids, as well as the need for proper labeling of these products, and the establishment and publications of guidelines in the handling of nicotine containing products, and addressing this as potential occupational hazard. Employees need to know. Given the high numbers of vape shops that have surfaced across the U.S., a large-scale education campaign may be warranted. The presence of vape shops on social media suggests that using online platforms in communication may be effective in delivering information to both consumers and vape shop employees^21,22^.

The research on tobacco retailer practices has shown that there are differences based on a community’s racial/ethnic profile^23–27^. One might speculate that we would see similar practices in the vape shop retail environment. Early research on vape shops has shown that there are some differences in tobacco product attitudes and tobacco product use based on racial/ethnic community^4^. The research presented here further investigated the presence of the differences in vape shop practices based on racial/ethnic community. The differences found in regards to training are also concerning in regards to how well informed vape shop employees are in different communities. It is important that vape shops be well informed given the recent FDA deeming rule that will make the practice of free samples illegal.

Much of the current public health focus on e-cigarettes and vape shops has pertained to flavorings, bans on use in public spaces, and access to minors,^2,28–31^ but the handling of nicotine-containing e-liquids in vape shops is also important. Currently, this area is largely self-regulated and the introduction of regulatory agency oversight would protect both the employees and the customers. Regulatory oversight could also help motivate vape shop owners/managers to provide appropriate training for their employees and proper protection for their customers against any potential harmful constituents found in e-liquids. There is evidence of vape shops responding well to public health policies or expected future policies, such as prohibiting sales to minors and prohibiting minors from entering shops^1^. It is imperative that regulations are put in place that protect vape shop employees and customers from exposure to nicotine in e-liquids.

### Limitations

Vape shops in Los Angeles may not be representative of shops in other areas of the U.S. Future studies should examine the handling of nicotine-containing e-liquids across the country. Comparing vape shops in states and cities with high regulatory environments to those with lower regulatory environments would inform federal agencies. Further, this study relied on a convenience sample based on Yelp searches and may not be representative of all vape shops and retailers. Future studies could build a sampling frame of vape shops based on business license records. A probability sampling procedure from this type of sampling frame would make samples more representative of vape shops in the LA area or greater US. It must also be noted, however, that this data was collected before the recent FDA deeming rule on e-cigarettes. Under the new regulations, free samples and trail puffs of e-cigarettes containing nicotine are forbidden. The data collection measures (interview and observation) did not specifically ask about the presences of warning labels on nicotine-containing products. This information would aid in understanding the effectiveness of such labels. Data were cross-section and longitudinal studies are needed to document how vape shops adapt to regulations in the future.

Despite these limitations, this study suggests the need for regulation in the handling of nicotine-containing e-liquids. Regulating the handling of nicotine by customers and vape shop employees is important to prevent unsafe practices and subsequent injury. Appropriate safety training and equipment should be provided to employees to prevent accidental exposure to nicotine. Findings should motivate the creation of other policies to protect both vape shop employees and customers from nicotine exposure, and provide strategies for more effective communication of potentially harmful constituents in e-liquids. Findings could also help in the creation of future educational materials and campaigns as well as new communication strategies from regulatory authorities.

## Figures and Tables

**Table 1:** Demographics (n=77)

Male	86%
Mean Age (yrs)	27.9 (sd=8.4)
Community Ethnicity	
Korean	23%
Hispanic	22%
African-American	26%
Non-Hispanic White	29%
Employee Ethnicity	
Asian	27%
Hispanic	9%
Non-Hispanic White	27%
Other	36%
Shop Position	
Owner	25%
Manager/Supervisor	39%
Clerk/Staff	33%
Mean Length of Employment (Months)	11.2 (sd=5.0)
Aware of FDA regulatory action regarding vape shops	95%
Sells disposable e-cigarettes	17%
Sells rechargeable e-cigarettes	100%

**Table 2:** Vape Shop Handling of Nicotine Containing E-Liquids (n=77)

Allows customers to sample e-liquids w/nicotine	50%
1–3 mg/mL	44%
6–10 mg/mL	85%
11–30 mg/mL	18%
Nicotine Containing E-Liquid Mixing	
Only sell pre-mixed (prior to arrival at shop)	56%
Mixed by shop employees	32%
Customer allowed to mix e-liquid	24%
Safety equipment provided to employees	84%
Provided for nicotine handling	56%
Provided for rebuilds	81%
Nicotine containing e-liquid spills	72%
Handles nicotine containing e-liquids without gloves	65%
Customer e-liquid self-service stations	83%

## References

[1] Sussman S, Baezconde-Garbanati L, Garcia R (2015). Commentary: Forces That Drive the Vape Shop Industry and Implications for the Health Professions. Eval Health Prof.

[2] Sussman S, Garcia R, Cruz TB, Baezconde-garbanati L, Pentz MA, Unger JB (2014). Consumers’ perceptions of vape shops in Southern California: an analysis of online Yelp reviews. Tob Induc Dis.

[3] Pearson A (2014). Local Strategies to Regulate Vape Shops & Lounges.

[4] Allem J-P, Unger JB, Garcia R, Baezconde-Garbanati L, Sussman S (2015). Tobacco Attitudes and Behaviors of Vape Shop Retailers in Los Angeles. Am J Health Behav.

[5] Bell K, Keane H (2012). Nicotine control: E-cigarettes, smoking and addiction. Int J Drug Policy.

[6] Richter P, Beistle D, Pederson L, O’Hegarty M (2008). Small-group discussions on menthol cigarettes: listening to adult African American smokers in Atlanta, Georgia. Ethn Health.

[7] Goniewicz ML, Gupta R, Lee YH (2015). Nicotine levels in electronic cigarette refill solutions: A comparative analysis of products from the U.S., Korea, and Poland. Int J Drug Policy.

[8] Kamerow D (2014). The poisonous “juice” in e-cigarettes. BMJ.

[9] Etter J-F, Zäther E, Svensson S (2013). Analysis of refill liquids for electronic cigarettes. Addiction.

[10] Cervellin G, Luci M, Bellini C, Lippi G (2013). Bad news about an old poison. A case of nicotine poisoning due to both ingestion and injection of the content of an electronic cigarette refill. Emerg Care J.

[11] FAULKNER JM (1933). NICOTINE POISONING BY ABSORPTION THROUGHTHESKIN. JAMA J Am Med Assoc.

[12] Basset RA, Osterhoudt K, Brabazon T (2014). Nicotine Poisoning in an Infant [Correspondence to the Editor]. N Engl J Med.

[13] Chatham-Stehens K, Law R, Taylor E (2014). Notes from the Field: Calls to Poison Centers for Exposures to Electronic Cigarettes — United States, September 2010–February 2014. MMWR Morb Mortal Wkly Rep.

[14] Willis HW (1937). Acute nicotine poisoning. J Pediatr.

[15] Ballard T, Ehlers J, Freund E, Auslander M, Brandt V, Halperin W (2010). Green Tobacco Sickness: Occupational Nicotine Poisoning in Tobacco Workers. Arch Environ Heal An Int J.

[16] Gehlbach SH, Williams WA, Freeman JI (1979). Protective Clothing as a Means of Reducing Nicotine Absorption in Tobacco Harvesters. Arch Environ Heal An Int J.

[17] Mayer B (2013). How much nicotine kills a human? Tracing back the generally accepted lethal dose to dubious self-experiments in the nineteenth century. Arch Toxicol.

[18] Arcury TA, Quandt SA Living and working safely: challenges for migrant and seasonal farmworkers. N C Med J.

[19] Richtel M Selling a Poison by the Barrel: Liquid Nicotine for E-Cigarettes. The New York Times.

[20] American Legacy Foundation Investigating Tobacco-Related Disparities.

[21] Cheney MK, Gowin M, Wann TF (2015). Vapor Store Owner Beliefs and Messages to Customers. Nicotine Tob Res.

[22] Chu K-H, Allem J-P, Cruz TB, Unger JB (2016). Vaping on Instagram: cloud chasing, hand checks and product placement. Tob Control.

[23] Henriksen L, Schleicher NC, Dauphinee AL, Fortmann SP (2012). Targeted advertising, promotion, and price for menthol cigarettes in California high school neighborhoods. Nicotine Tob Res.

[24] Dauphinee AL, Doxey JR, Schleicher NC, Fortmann SP, Henriksen L (2013). Racial differences in cigarette brand recognition and impact on youth smoking. BMC Public Health.

[25] Yerger VB, Przewoznik J, Malone RE (2007). Racialized geography, corporate activity, and health disparities: tobacco industry targeting of inner cities. J Health Care Poor Underserved.

[26] Widome R, Brock B, Klein EG, Forster JL (2012). Smokeless tobacco advertising at the point of sale: prevalence, placement, and demographic correlates. Nicotine Tob Res.

[27] Widome R, Brock B, Noble P, Forster JL (2013). The relationship of neighborhood demographic characteristics to point-of-sale tobacco advertising and marketing. Ethn Health.

[28] Carr ER (2014). E-Cigarettes: Facts, Perceptions, and Marketing Messages - ProQuest. Clin J Oncol Nurs.

[29] Cheney M, Gowin M, Wann TF (2015). Marketing Practices of Vapor Store Owners. Am J Public Health.

[30] Cheney MK, Gowin M, Wann TF (2015). Vapor Store Owner Beliefs about Electronic Cigarette Regulation. Tob Regul Sci.

[31] Lee YO, Kim AE (2014). “Vape shops” and “E-Cigarette lounges” open across the USA to promote ENDS. Tob Control.

